# Memory Impairment in Estrogen Receptor α Knockout Mice Through Accumulation of Amyloid-β Peptides

**DOI:** 10.1007/s12035-014-8853-z

**Published:** 2014-08-17

**Authors:** Chul Ju Hwang, Hyung-Mun Yun, Kyung-Ran Park, Ju Kyung Song, Hyun Ok Seo, Byung Kook Hyun, Dong Young Choi, Hwan-Soo Yoo, Ki-Wan Oh, Dae Yeun Hwang, Sang-Bae Han, Jin Tae Hong

**Affiliations:** 1 College of Pharmacy and Medical Research Center, Chungbuk National University, 52, Naesudong-ro, Heungdeok-gu, Cheongju, Chungbuk 361-763 Republic of Korea; 2 College of Pharmacy, Young Nam University, Dae, Gyeongsan, Gyeongbuk 712-749 Republic of Korea; 3 College of Natural Resources & Life Science, Pusan National University, Pusan, Republic of Korea; 4 College of Pharmacy, Medical Research Center, Chungbuk National University, 12, Gaeshin-dong, Heungdeok-gu, Cheongju, Chungbuk 361-763 Republic of Korea

**Keywords:** Alzheimer’s disease, Amyloid, Estrogens, Estrogen receptor alpha, Neprilysin, Neurogenic inflammation

## Abstract

Estrogen has been known to reduce the development of Alzheimer’s disease (AD). However, exact mechanisms are not clear. We investigated whether estrogen can increase amyloid-beta (Aβ) degradation and affects Aβ-induced memory impairment in an estrogen deficiency model. Estrogen receptor alpha (ERα) knockout mice and wild-type mice were intracerebroventricular (ICV) infused with Aβ (300 pmol) for 2 weeks. Cognitive function was then assessed by the Morris water maze test and passive avoidance test. In addition, Western blot analysis, immunostaining, immunofluorescence staining, ELISA, and enzyme activity assays were used to examine the degree of Aβ deposition in the brains of ERα knockout mice. In our present study, Aβ was accumulated more in the ERα knockout mice brain and greatly worsened memory impairment and glial activation as well as neurogenic inflammation. These results suggest that estrogen may protect memory impairment by stimulating the degradation of Aβ and down-regulate neurogenic inflammation as well as amyloidogenesis.

**Electronic supplementary material:**

The online version of this article (doi:10.1007/s12035-014-8853-z) contains supplementary material, which is available to authorized users.

## Introduction

Estrogen is important in the maintenance of normal brain function. It interacts with many different receptors including alpha and beta estrogen receptors, both of them highly expressed in the hippocampus and cortex, the two brain regions most implicated in the develop of Alzheimer’s disease (AD) [1]. Reduced estrogen levels due to aging increases the risk of AD [2]. The Women’s Health Initiative found that estrogen therapy on cognitive function for post-menopausal women is no longer beneficial for treatment [3, 4]. However, recent data indicate that estrogen therapy may be effective if treated during the perimenopausal period [5]. It was also found that estrogen is effective in lowering the level of brain amyloid-beta (Aβ), a causing factor for the development of AD in ovariectomized transgenic mice that overexpress Aβ [6]. Estrogen was also reported to reduce the generation of Aβ peptides in cells and neurons [7]. Thus, the lowering effect of estrogen on Aβ levels in the brain could be a possible mechanism of its memory improving effect. However, clear mechanisms of how estrogen lowers Aβ levels are not defined.

Neprilysin is thought to be the primary Aβ-degrading enzyme in the brain because degradation of radiolabeled synthetic Aβ_42_ in rat brain is largely inhibited by the neprilysin inhibitor, phosphoramidon. Neprilysin degrades both monomeric and oligomeric forms of Aβ_40_ and Aβ_42_ in intracellular and extracellular compartments of the brain [8]. Moreover, the levels of neprilysin mRNA and protein are lower in the hippocampus and temporal gyrus of AD patients [9, 10]. Neprilysin activity is also lower in the hippocampus, cerebellum, and caudate of ovaritectomized rats than in non-ovariectomized rats, and this effect can be reversed by exogenous 17β-estradiol [11]. Furthermore, two functional estrogen response elements (EREs) were identified in the neprilysin gene, which bind estrogen receptor alpha (ERα) and ERβ and thus stimulate ER-dependent gene expression [12]. These data provide insight into the positive effects of estrogen on neprilysin activity in the brain and thus affect Aβ levels for improving cognitive performance in menopausal women.

There has been a significant association between expressions of ERα or ERβ and memory function. However, ERα is more functional for the etiology of memory, and ERα-mediated behaviors are more sensitized to estrogen in humans and mice [12]. In this study, we investigated whether the knockout of ERα could down-regulate neprilysin and other Aβ-degrading enzyme expressions and activities, thus diminishing Aβ-scavenging activity leading to the enhancement of neuronal cell death and memory impairment in Aβ_42_-infused ERα knockout mice.

## Methods

### Aβ_1–42_-Infused ERα Knockout Mice

ERα knockout mice were purchased from The Jackson Laboratory (Bar Harbor, Maine, USA). All of the experimental procedures were approved by the Animal Care and Use Committee (IACUC) of Chungbuk National University (approval number: CBNUA-144-1001-01). Four experimental groups of mice (*n* = 8) were studied: the C57BL/6 wild-type (control) group (saline-infused = 8, Aβ-infused = 8) and the ERα knockout mice (saline-infused = 8, Aβ-infused = 8). All were female weighing 24–28 g (age 8–10 weeks old) at the time of exposure. ERα knockout mice grow and reproduce normally with no obvious phenotypic difference from the wild type. All mice were housed in a room that was automatically maintained at 21–25 °C and relative humidity (45–65 %) with a controlled light–dark cycle. The lights were on at 0600 h until 1800 h (KST).

The infusion model was adapted from previous work on the mice infusion model [13]. The anesthetized mice were placed in a sterotaxic instrument, and catheters were attached to an osmotic mini-pump (Alzet 1002, ALZA, Mountain View, CA, USA) and brain infusion kit 1 (Alzet kit 3–5 mm, ALZA, Mountain View, CA, USA) which were implanted according to the following coordinates: mouse (unilaterally) −1.0 mm anterior/posterior, +0.5 mm medial/lateral, and −2.5 mm dorsal/ventral. The pump contents were released over a period of 2 weeks consisting of 300 pmol aggregated (the lyophilized peptide of Aβ_1–42_ is dissolved in DW and incubated at 37 °C for 48 h) Aβ_1–42_ (Bachem Chemical, Kashiwa St, Torrance, CA, USA) dissolved in sterile saline (0.9 % NaCl) for each pump. The pump of the control group contained saline (0.9 % NaCl) for each pump.

### Morris Water Maze Test

The Morris water maze test is a widely accepted method for examining cognitive function and was used in the present study as previously described [14]. Briefly, a circular plastic pool (height 35 cm and diameter 100 cm) was filled with water (plus white dye) maintained at 22–25 °C. An escape platform (height 14.5 cm and diameter 4.5 cm) was submerged 1–1.5 cm below the surface of the water. The test was performed three times a day for 6 days during the acquisition phase (days 1–6), with three randomized starting points. The position of the escape platform was kept constant. Each trial lasted for 60 s or ended as soon as the mice reached the submerged platform. The swimming pattern of each mouse was monitored and recorded by a camera mounted above the center of the pool, and the escape latency, escape distance, and swimming speed were assessed by the SMART-LD program (Panlab, Barcelona, Spain). A quiet environment, consistent lighting, constant water temperature, and a fixed spatial frame were maintained throughout the experimental period.

### Probe Test

To assess memory consolidation, a probe test was performed 48 h after the water maze test (i.e., day 8). For the probe test, the platform was removed from the pool, and the mice were allowed to swim freely. The swimming pattern of each mouse was monitored and recorded for 60 s using the SMART-LD program (Panlab). Consolidated spatial memory was estimated by the time spent in the target quadrant area.

### Passive Avoidance Test

The passive avoidance response was determined using a “step-through” apparatus (Med Associates, Georgia, VT, USA). Forty-eight hours after the probe test (i.e., day 10), a training trial was performed. After the training trial, each mouse was placed in the illuminated compartment of the apparatus facing away from the dark compartment. When the mouse moved completely into the dark compartment, it received an electric shock (0.4 mA, 3-s duration). Twenty-four hours after the training trial (i.e., day 11), each mouse was placed in the illuminated compartment, and the latency period until it entered the dark compartment was determined and defined as the step-through latency. The cutoff time for the examination was 180 s.

### Collection and Preservation of Brain Tissues

After the passive avoidance test, mice were anesthetized with diethyl ether and then perfused with phosphate-buffered saline (PBS). The brains were immediately removed from the skull, and the cortex and hippocampus were dissected on ice. All brain tissues were stored at −80 °C until biochemical analysis.

### Immunohistochemical Staining

After being anesthetized with diethyl ether, subgroups of mice were perfused intracardially with 50 ml saline. The brains were taken out from the skull and post-fixed in 4 % paraformaldehyde for 24 h at 4 °C. The brains were transferred to 30 % sucrose solutions. Subsequently, brains were cut into 25-μm sections by using a cryostat microtome (Leica CM1850; Leica Microsystems, Seoul, Korea). After multiple washes in PBS, endogenous peroxidase activity was quenched by incubating the samples in 3 % hydrogen peroxide in PBS for 30 min, followed by a 10-min wash in PBS. The sections were then incubated for 2 h at room temperature with a rabbit/mouse polyclonal antibody against glial fibrillary acidic protein (GFAP), inducible nitric oxide synthase (iNOS), receptor for advanced glycation end products (RAGE) (1:300; Abcam, Inc, Cambridge, MA, USA), a rabbit polyclonal antibody against cyclooxygenase-2 (Cox-2), and Aβ (1:300; Cell Signaling Technology, Inc., Beverly, MA, USA). After incubation with the primary antibodies, sections were washed in PBS before being incubated for 1 h at room temperature in the presence of biotinylated goat anti-rabbit or anti-mouse IgG secondary antibodies (1:1,000; Vector Laboratories, Burlingame, CA, USA). Sections were then washed with PBS and incubated with avidin–peroxidase complex (Vector Laboratories, Burlingame, CA, USA) for 30 min before the immunocomplex was visualized using the chromogen 3,3′-diaminobenzidine (Vector Laboratories, Burlingame, CA, USA). Sections were then counterstained with hematoxylin. Finally, sections were dehydrated in ethanol, cleared in xylene, and covered with Permount. Immunohistochemical staining was performed on eight mice per group.

### Western Blot Analysis

The brain and liver tissues were homogenized with lysis buffer (PRO-PREP; iNtRON, Seongnam, Korea; *n* = 8 mice per group) and centrifuged at 2,500×*g* for 15 min at 4 °C. Equal amounts of total protein (40 μg) isolated from the hippocampus regions of brain tissues were resolved on 8 or 10 % sodium dodecyl sulfate polyacrylamide gels and then transferred to nitrocellulose membranes (Hybond ECL; Amersham Pharmacia Biotech, Piscataway, NJ, USA). Membranes were incubated at room temperature for 2 h with the following specific antibodies: anti-GFAP, anti-RAGE (both 1:1,000; Abcam, Inc, Cambridge, MA, USA), anti-Cox-2, anti-β-secretase (BACE) 1 (Cell Signaling Technology, Inc., Beverly, MA, USA), anti-NEP, anti-iNOS, anti-amyloid precursor protein (anti-APP) (1:1,000, Novus Biologicals, Inc., Littleton), anti-BAX, anti-caspase-3, anti-lipoprotein receptor-related protein (LRP), anti-matrix metalloproteinase (MMP)-9 (1:1,000; Santa Cruz Biotechnology, Inc., Santa Cruz, CA, USA), and anti-β-actin (1:2,500; Sigma, St Louis, MO, USA). Blots were then incubated at room temperature for 2 h with corresponding peroxidase-conjugated anti-mouse or anti-rabbit antibodies (1/2,000; Santa Cruz Biotechnology, Inc., Santa Cruz, CA, USA). Immunoreactive proteins were detected using an enhanced chemiluminescence [7] Western blotting detection system. The relative density of the protein bands was scanned densitometrically using My Image (SLB, Seoul, Korea) and quantified by Lab Works 4.0 (UVP, Upland, CA, USA).

### Measurement of Aβ_1–40_ and Aβ_1–42_

Lysates of brain tissue were obtained through protein extraction buffer containing protease inhibitor. Aβ_1–40_ and Aβ_1–42_ levels were determined using each specific ELISA Kit (Immuno-Biological Laboratories Co., Ltd., Takasaki-Shi, Gunma, Japan). In brief, 100 μl of sample was added into a precoated plate and incubated overnight at 4 °C. After washing each well of the precoated plate with a washing buffer, 100 μl of labeled antibody solution was added, and the mixture was incubated for 1 h at 4 °C in the dark. After washing, chromogen was added, and the mixture was incubated for 30 min at room temperature in the dark. Finally, the resulting color was assayed at 450 nm using a microplate absorbance reader (Sunrise™, Tecan, Switzerland) after adding stop solution.

### Thioflavin S Staining

The brains were taken out from the skull and post-fixed in 4 % paraformaldehyde for 24 h at 4 °C. The brains were transferred to 30 % sucrose solutions. Subsequently, brains were cut into 25-μm sections by using the cryostat microtome (Leica CM1850; Leica Microsystems, Seoul, Korea). The sections of the brain were thoroughly washed with distilled water for 5 min and then transferred to gelatin-coated slices and placed in 1 % thioflavin S for 5 min. After this, the sections were washed in distilled water then dehydrated through ascending grades of ethanol, 50, 70, 90, and 100 % ethanol for 2 min in each grade. The sections were then mounted in a mounting medium (Fluoromount™ Aqueous Mounting Medium, Sigma, St Louis, MO, USA). The thioflavin S staining was examined using a fluorescence microscope. Thioflavin staining was performed on eight mice per group.

### Assay of Neprilysin Activities

Five randomly chosen mice from each group were examined. Neprilysin activities were determined using a neprilysin activity assay kit (CBA079, Calbiochem, Darmstadt, Germany) according to the manufacturer’s protocols. Briefly, brains (hippocampus regions) were homogenized in 50-mM potassium phosphate buffer, pH 7.3, containing a proteinase inhibitor mix (Sigma, St Louis, MO, USA). Samples were centrifuged, and the supernatant fraction was used for neprilysin activity measurement. The hydrolysis of fluorogenic substrate peptides (2 mM Mca-RPPGFSAFKDNP-OH, R&D system, no. ES005) in 20-mM potassium phosphate buffer, pH 7.3) was measured by following an increase in fluorescence (exCitation at 320 nm and emission at 405 nm) that occurred upon peptide bond cleavage for neprilysin activity. The neprilysin activities were indicated as nanomole (obtained from a standard curve using a fluorescence unit) per microgram of protein per minute.

### Assay of β-Secretase Activities

β-Secretase activity in mice brains was determined using a commercially available β-secretase activity kit (Abcam, Inc, Cambridge, MA, USA). Protein was extracted from brain tissues (hippocampus regions) using ice-cold extraction buffer, incubated on ice for 20 min and centrifuged at 10,000×*g* for 5 min at 4 °C. The supernatant was collected. A total of 50 μl of sample (total protein 100 μg) was added to each well followed by 50 μl of 2 × reaction buffer and 2 μl of beta-secretase substrate incubated in the dark at 37 °C for 2 h. Fluorescence was read at excitation and emission wavelengths of 355 and 510 nm, respectively, using a Fluostar Galaxy fluorimeter (BMG Lab Technologies, Offenburg, Germany) with Felix software (BMG Lab Technologies, Offenburg, Germany). Beta-secretase activity is proportional to the fluorimetric reaction and is expressed as nanomole per milligram of protein per minute.

### Cresyl Violet Staining

The brains were taken out from the skull and post-fixed in 4 % paraformaldehyde for 24 h at 4 °C. The brains were transferred to 30 % sucrose solutions. Subsequently, brains were cut into 25-μm sections by using the cryostat microtome (Leica CM1850; Leica Microsystems, Seoul, Korea). The brain sections were thoroughly washed with PBS to remove the excess fixative agent and then transferred to gelatin-coated slices and stained with 0.1 % cresyl violet (2–5 min) for the purpose of identifying cortical layers and cytoarchitectural features of the isocortical region. After this, the sections were washed in distilled water and then dehydrated through ascending grades of ethanol, 50, 70, 90, and 100 % ethanol for 2 min in each grade followed by a 10-min immersion in a 1:1 mixture of absolute alcohol and xylene. They were cleared in xylene for 5–10 min and mounted in a mounting medium (CYTOSEAL™ XYL; Thermo Scientific, Pittsburgh, CO, USA). Cresyl violet staining was performed on eight mice per group. For tissue measurements and counting, the tissues were photographed in the same areas. The positive neurons in each section were manually counted per 1-mm length area under ×200 magnification by light microscopy.

### Statistical Analysis

All statistical analysis was performed with GraphPad Prism 4 software (Version 4.03; GraphPad software, Inc., San Diego, CA, USA). Group differences in the escape distance, latency, and velocity in the Morris water maze task were analyzed using *t* test repeated measures, the factors being treatment and testing day. Otherwise, differences were analyzed by two-way ANOVA followed by Dunnett’s post hoc test. All values are presented as mean ± S.E.M. Significance was set at *P* < 0.05 for all tests.

## Results

### Effect of Knockout of ERα on Memory Impairment

**Fig. 1:**
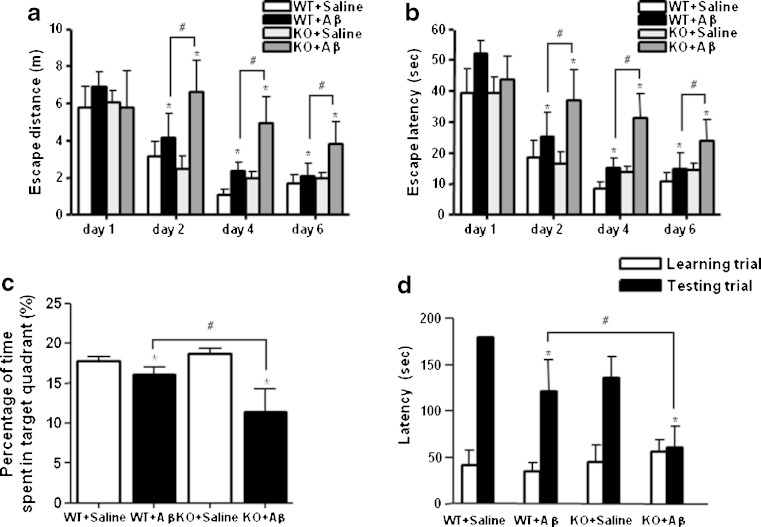
Effect of ERα knockout on memory impairment, Training trial was performed three times a day for 6 days. Swimming distance (**a**) and swimming time (**b**) to arrive at the platform were automatically recorded. One day after training trials, a probe test was performed. The time spent in the target quadrant and target site crossing within 60 s were represented (**c**). To perform the passive avoidance test, the mice were given electric shock when they entered into the dark compartment for training on learning day. After 1 day, the retention time in illuminated step-through test compartment was recorded (**d**). Each value is presented as mean ± S.E.M. from eight mice. An *asterisk* indicates a significant difference between saline-infused and Aβ-infused mice (*P* < 0.05). A *number sign* indicates a significant difference between Aβ-infused C57BL/6 wild-type mice and Aβ-infused ERα KO mice (*P* < 0.05)

Knockout of ERα mice and C57BL/6 wild-type mice was infused with Aβ_1–42_ (300 pmol/day/mouse) for 2 weeks and then compared for memory deficiency with saline-infused C57BL/6 wild-type mice using the Morris water maze and passive avoidance tests. All mice were trained for three trials per day for 6 days. Escape latency and escape distances which are the time and distance travelled to reach platform in water maze were measured to determine the effect of knockout of ERα on memory impairment. The mice exhibited shorter time and shorter escape latency through the training; however, the escape latency of Aβ_1–42_-infused mice in both knockout of ERα mice and C57BL/6 wild-type mice was not significantly reduced compared to the saline-infused C57BL/6 wild-type mice, especially in knockout of ERα mice. Statistical analysis of data from days 1, 2, 4, and 6 showed that Aβ_1–42_-infused knockout of ERα mice has more memory impairment than Aβ_1–42_-infused C57BL/6 wild-type mice. Escape distance at the fourth and sixth day of the Aβ_1–42_-infused knockout of the ERα mice group was longer than that of the Aβ_1–42_-infused C57BL/6 wild-type mice group (Fig. 1a). Escape latency was also increased in the Aβ_1–42_-infused knockout of the ERα mice group (Fig. 1b). However, there was no significant difference in average speed between the Aβ_1–42_-infused knockout of the ERα mice group and the Aβ_1–42_-infused C57BL/6 wild-type mice group (data not shown).

After the water maze test, we performed a probe test to investigate the maintenance of memory. However, the time spent in the target quadrant by infusion of Aβ_1–42_ has decreased in knockout of the ERα mice group (16.24 ± 1.08 s) compared with the Aβ_1–42_-infused C57BL/6 wild-type mice (11.42 ± 4.25 s) during the probe test. The probe test of the Aβ_1–42_-infused knockout of the ERα mice group was shorter than that of the Aβ_1–42_-infused C57BL/6 wild-type mice group (Fig. 1c).

We next evaluated the learning and memory capacities by the passive avoidance test using the step-through method. The passive avoidance test of the Aβ_1–42_-infused knockout of the ERα mice group was shorter than that of the Aβ_1–42_-infused C57BL/6 wild-type mice group (Fig. 1d). There was no significant difference in the learning trial. However, in the test trial, infusion of Aβ_1–42_ decreased the step-through latency, and infusion with Aβ_1–42_ (60.89 ± 22.4 s) in the knockout of ERα mice greatly decreased the step-through latency compared with the Aβ_1–42_-infused C57BL/6 wild-type mice (121.2 ± 33.58 s).

### Effect of ERα Knockout on Cell Death in ERα Knockout Mouse Brain

**Fig. 2:**
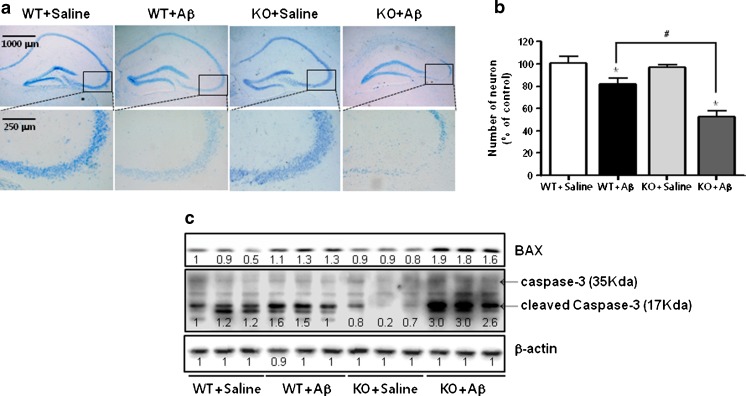
Effect of ERα knockout on cell death in a mouse brain. Neuronal cells were stained with cresyl violet (**a**). The graph represents quantitation of the number of neuronal cells (**b**). Total cell number counted × 200. All values are the means ± S.E. from three mice brains. An *asterisk* indicates a significant difference between saline-infused to Aβ-infused mice (*P* < 0.05). A *number sign* indicates a significant difference between Aβ-infused C57BL/6 wild-type mice and Aβ-infused ERα KO mice (*P* < 0.05)

Cresyl violet staining was used to examine the survival neurons. Neuronal cells were stained with cresyl violet clearly in saline-infused C57BL/6 mice, but not well defined in Aβ_1–42_-infused C57BL/6 wild-type mice and ERα knockout mice (Fig. 2a). The numbers of viable neurons per 1-mm length of cornu ammonis 3 (CA3) neuronal cells (% of cresyl-violet-stained area) were quantitatively analyzed. Survival of neuronal cells (51.93 ± 1.67 %) in knockout of ERα mice greatly decreased compared with the Aβ_1–42_-infused C57BL/6 wild-type mice (82.34 ± 2.4 %) (Fig. 2b). Western blot data for BAX and caspase-3(a pro-apoptotic protein) expression (Fig. 2c) were also increased in Aβ_1–42_-infused ERα knockout mice brain compared to the expression in Aβ_1–42_-infused C57BL/6 wild-type mice brain.

### Enhancement Effect of ERα Knockout on Aβ Accumulation and Amyloidogenesis

**Fig. 3:**
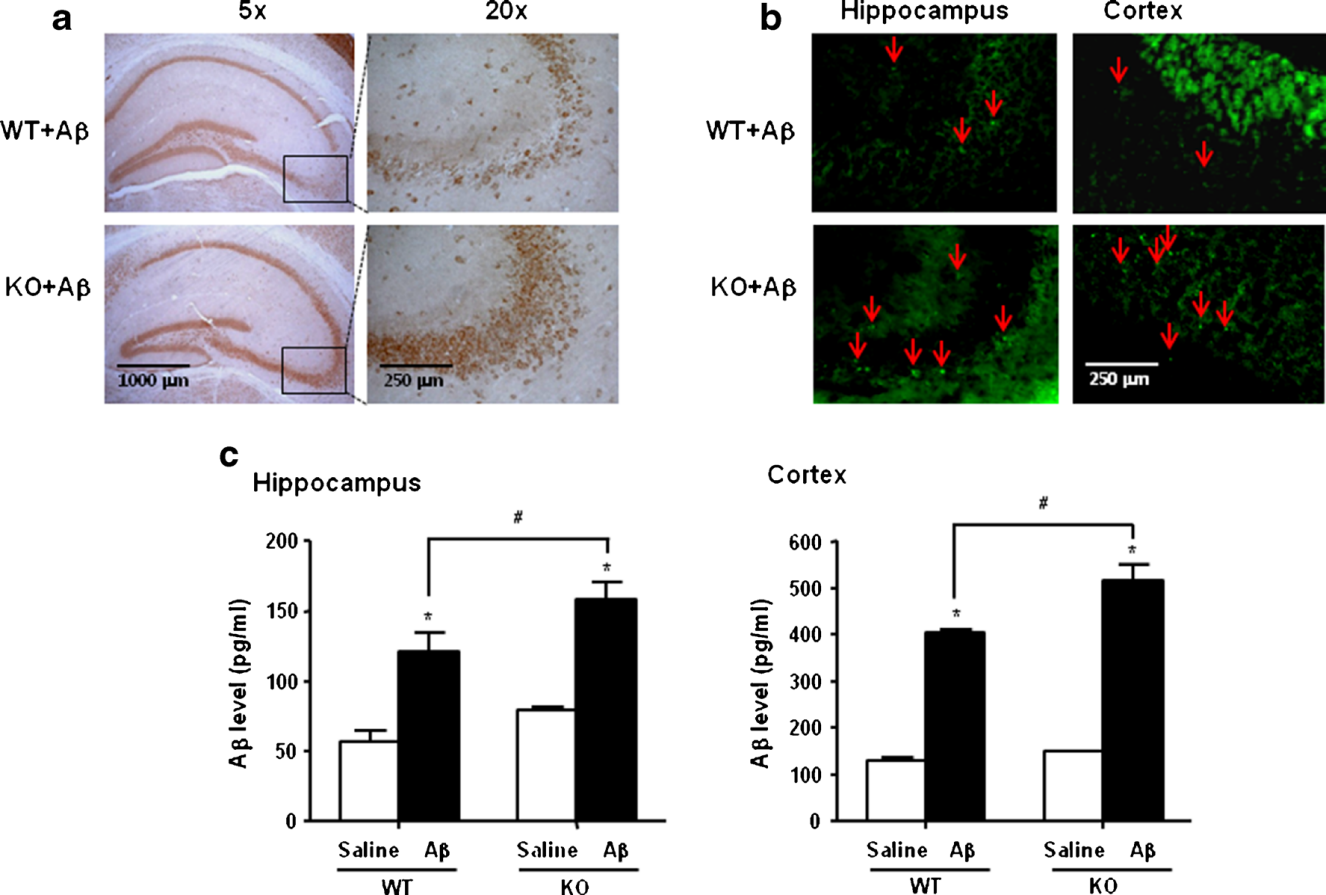
Effect of ERα knockout on the Aβ accumulation and Aβ level in mouse brain. Immunostaining of amyloid-β protein in the hippocampus was performed in 30-μm-thick sections of ERα knockout brain with anti-Aβ_1–42_ primary antibody and the biotinylated secondary antibody (**a**). Thioflavin S staining for detection of Aβ accumulation. The *arrow* in the graph represents immunoreactive β-amyloid reactive cells in the mice brain (**b**). Inhibitory effect of ERα knockout on the level of Aβ_1–42_ in brain hippocampus (**c**) and cortex (**d**) detected by the ELISA method. Values were mean ± S.D. of eight mice. An *asterisk* indicates a significant difference between saline-infused and Aβ-infused mice (*P* < 0.05). A *number sign* indicates a significant difference between Aβ-infused C57BL/6 wild-type mice and Aβ-infused ERα KO mice (*P* < 0.05)

Several studies reported that the accumulation of Aβ in the brain is the major causing factor in the development of AD. So, we examined Aβ accumulation in both Aβ_1–42_-infused ERα knockout mice and C57BL/6 wild-type mice. The immunohistochemical analysis by an Aβ_42_-specific antibody has shown the Aβ deposition in the cortex and hippocampus regions of Aβ_1–42_-infused ERα knockout mice and C57BL/6 wild-type mice. However, accumulation of Aβ in the brain of Aβ_1–42_-infused ERα knockout mice was much higher compared to the Aβ_1–42_-infused C57BL/6 wild-type mice brain (Fig. 3a). In addition, we confirmed the higher accumulation of Aβ in the brains of Aβ_1–42_-infused ERα knockout mice by thioflavin S staining (Fig. 3b). To determine whether Aβ deposition by immunohistochemical analysis was paralleled with Aβ protein level in the brain tissue, quantitative analyses of Aβ_1–42_ levels were performed using an Aβ_1–42_-specific ELISA kit. Aβ_1–42_ levels in the brains of Aβ_1–42_-infused ERα knockout mice were significantly higher compared to the levels of Aβ_1–42_ in the Aβ_1–42_-infused C57BL/6 wild-type mice brain (Fig. 3c). In addition, to evaluate BACE activity, we performed Western blot analysis APP and BACE1 and β-secretase activity assay using a commercially available β-secretase activity kit. Western blot analysis also revealed that APP and BACE1 expression were significantly higher in the brains of Aβ_1–42_-infused ERα knockout mice compared to Aβ_1–42_-infused C57BL/6 wild-type mice (Supplementary Fig. 2a). And, β-secretase activity was significantly increased by the absence of ERα in the Aβ_1–42_-infused brain (Supplementary Fig. 2b).

### Effect of ERα Knockout on the Expression of Cox-2, iNOS, and GFAP

**Fig. 4:**
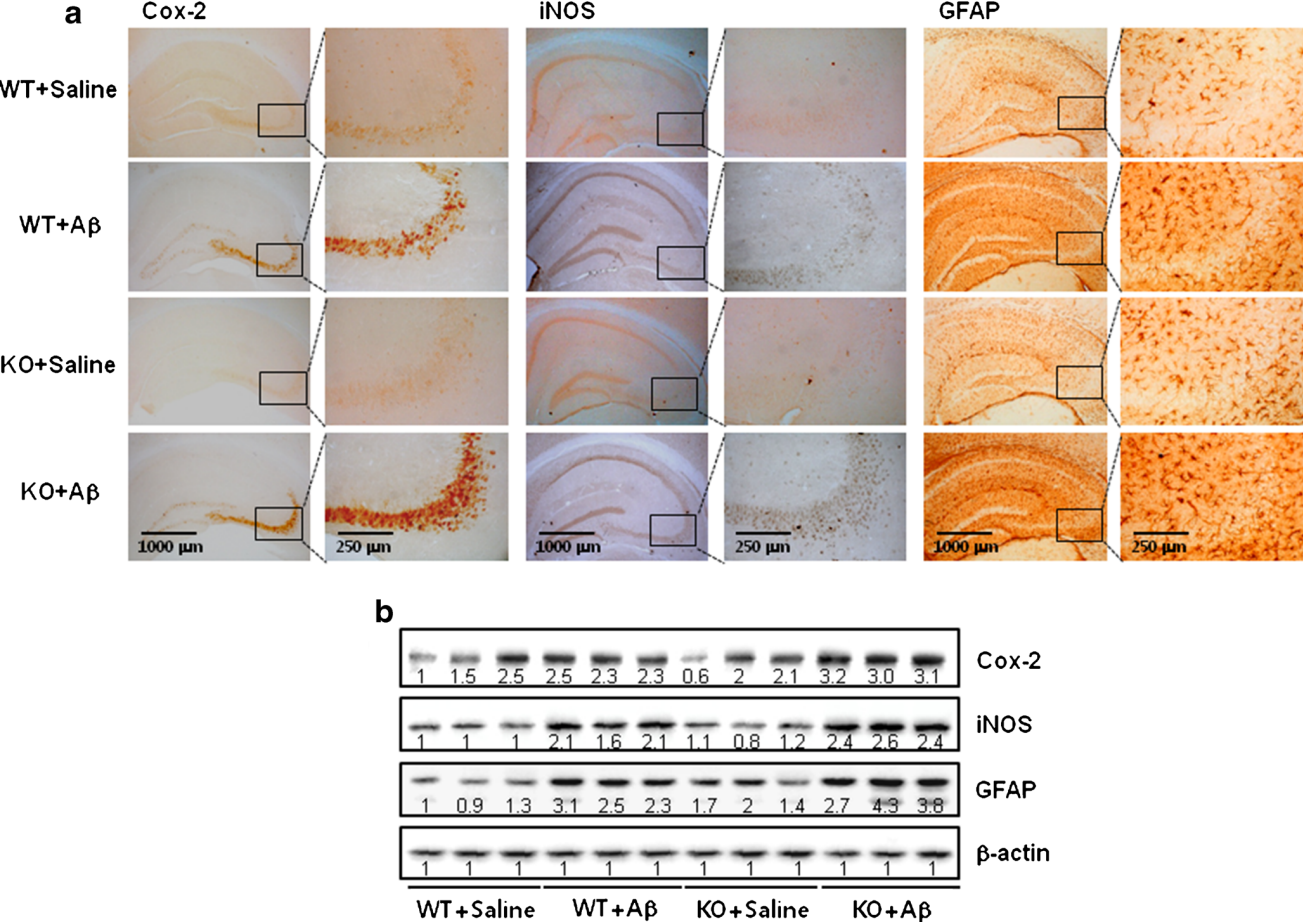
Effect of ERα knockout on the activation of astrocytes and the expression of Cox-2 and iNOS in mouse brain. The sections of mouse brain incubated with anti-GFAP, Cox-2, and iNOS primary antibody and the biotinylated secondary antibody (*n* = 3). The representative stained tissues were viewed with a microscope (×50 or 200) (**a**). Expression of GFAP, Cox-2, and iNOS were also examined by specific antibodies in the brain hippocampus (**b**). Each blot is representative for three mice

We previously found that neuroinflammation is critical for Aβ generation, and astrocytes are important neuronal cells contributing to neuroinflammation and amyloidogenesis. To determine whether ICV administration with Aβ can also induce neuroinflammation, and activation of astrocytes, we used the methods of Western blot and immunohistochemistry to detect the expression of Cox-2, iNOS, and GFAP in mouse brains (Fig. 4a, b). Our data indicate that Aβ_1–42_-infused ERα knockout mice significantly increase these proteins’ expression in the hippocampus and cortex when compared with Aβ_1–42_-infused C57BL/6 wild-type mice. Immunostaining for Cox-2, iNOS, and GFAP (a marker of astrocyte activation) (Fig.4a) also showed a significantly higher number of Cox-2, iNOS, and GFAP reactive cells in Aβ_1–42_-infused ERα knockout mice brain compared to the number in Aβ_1–42_-infused C57BL/6 wild-type mice brain.

### Effect of ERα Knockout on the Expression of Neprilysin and Activities of Neprilysin

**Fig. 5:**
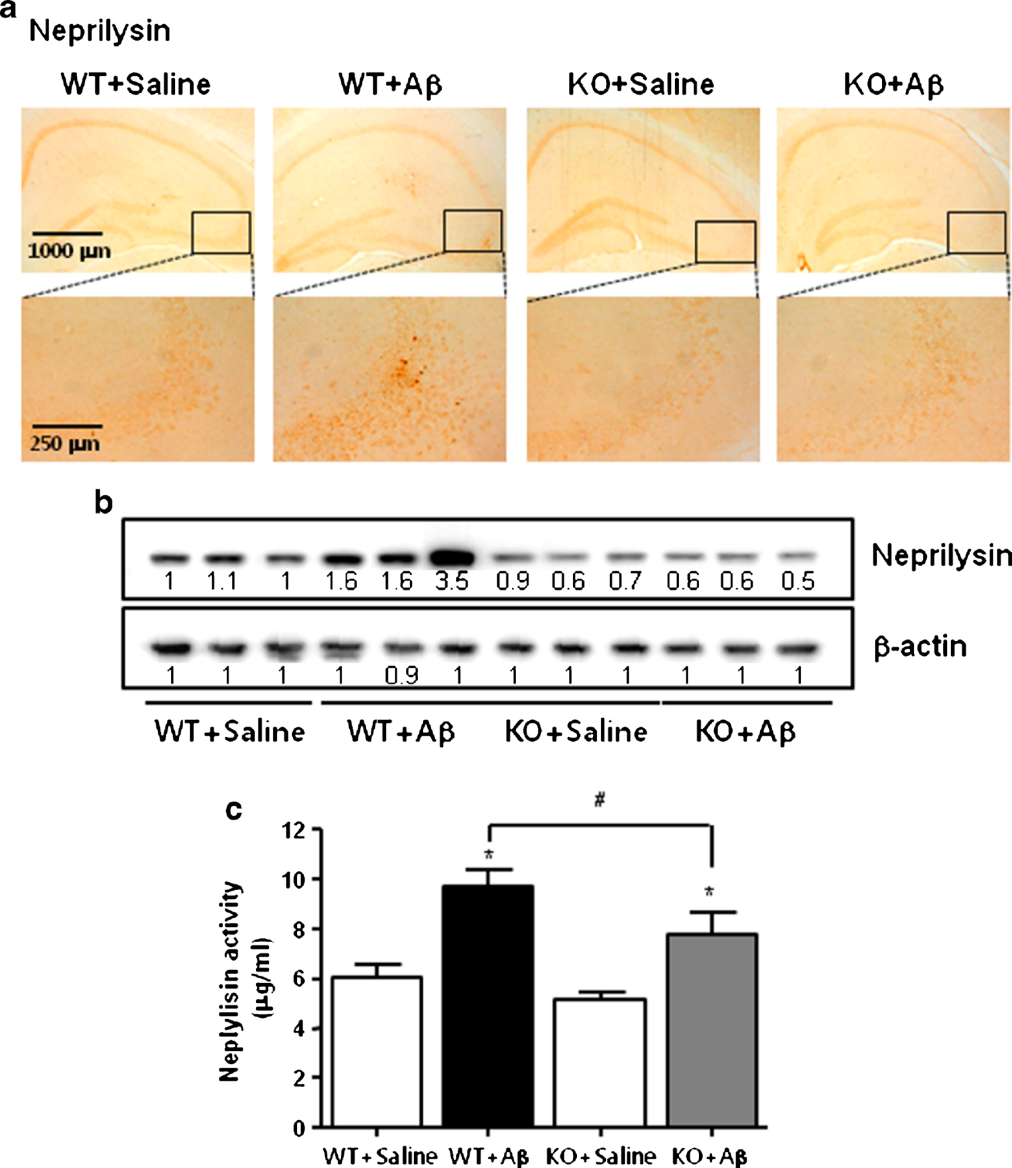
Effect of ERα knockout on the expression and activity of neprilysin in mouse brain. The representative stained tissues were viewed with a microscope (×50 or 200) (**a**). Tissue lysates from mouse brain were probed with Aβ-degrading peptidases, neprilysin protein antibodies, respectively. Experiments were performed with three mouse brains (**b**). Neprilysin activities were measured in the brains as mentioned in the “Material and Methods”. All values are the means ± S.E. from eight mice brains (**c**). An *asterisk* indicates a significant difference between saline-infused and Aβ-infused mice (*P* < 0.05). A *number sign* indicates a significant difference between Aβ-infused C57BL/6 wild-type mice and Aβ-infused ERα KO mice (*P* < 0.05)

We studied the activity of Aβ-degrading peptidase neprilysin for studying possible causes of elevated levels of Aβ_1–42_ in the Aβ_1–42_-infused ERα knockout mice brain since neprilysin has an estrogen response element promoter region [15]. Decreased neprilysin reactive cell number (Fig. 5a) and expressions of neprilysin (Fig. 5b) were found in Aβ_1–42_-infused ERα knockout mice brain. Agreement with enhanced expression of these proteins, the activities of neprilysin were also significantly decreased in Aβ_1–42_-infused ERα knockout mice brain (Fig. 5c). The accumulation of Aβ_1–42_ can also be influenced by the influx of Aβ_1–42_ into the brain through a RAGE in the brain. We determined RAGE expression by immunohistochemical analysis (Supplementary Fig. 1a) and by Western blotting (Supplementary Fig. 1b). But, expressions of RAGE between the two groups are unclear.

### Effect of ERα Knockout on the Expression of MMP-9 and LRP-1 in ERα Knockout Mouse Liver

Since liver is another tissue involving systemic elimination of Aβ_1–42_ in the blood through LRP-1, we determined LRP-1 in the liver. MMP-9 is also critical for the degradation of Aβ in the liver, so we also detected MMP-9 expression. Expression of LRP-1 and MMP-9 was lowered in the Aβ_1–42_-infused ERα knockout mice liver (Supplementary Fig. 1c). These data indicate that a decreased degradation and/or clearance of Aβ_1–42_ in the liver are related with increased Aβ_1–42_ accumulation in an Aβ_1–42_-infused ERα knockout mice brain.

## Discussion

Our present data showed that an intracerebroventricular infusion of Aβ_1–42_ caused worsened memory impairment and amyloidogenesis as well as neuroinflammation in ERα knockout mice compared to the Aβ_1–42_-infused C57BL/6 wild-type mice. The worsened memory impairment in the ERα knockout mice was associated with elevated Aβ level, astrogliosis and neuroinflammation, and apoptotic neuronal cell death in the brain. It was also found that the expression and activities of neprilysin in the brain and expression of LRP and MMP-9 in the liver of the ERα knockout mice were significantly lowered. These data indicate memory impairment in the ERα knockout mice through accumulation of Aβ via reduced degradation and scavenging of Aβ by the reduced expression and activity of neprilysin. Thus, it is clear that knockout of ERα promotes worsened memory function.

Several ways could be explained as to the possible mechanisms in the worsened memory impairment in ERα knockout mice. The accumulation of Aβ in the brain may be a significant contributor in the memory impairment in the ERα knockout mice. A hypothesis that Aβ accumulation is closely associated with the development of AD in animal models as well as human patients [16, 17], we found significantly higher accumulation of Aβ in the brains of ERα knockout mice. Thus, the higher accumulation of Aβ in the brain of ERα knockout mice could result in worsened memory dysfunction. Interestingly, the present study also showed that cell death, Aβ accumulation, and neuroinflammation were clearer in the CA3 region than in other brain regions. Recent study indicated that estrogen effects on hippocampal neurons are more pronounced in the CA3 region. There is also higher expression of ERα in the CA3 region than in other regions of the hippocampus including CA1 and DG [18]. This evidence supported the more significant effect of estrogen on the CA3 region.

Accumulation of Aβ_1–42_ levels is due to decreased Aβ clearance through the inhibition of the degradation in the brain and/or decrease of efflux from the brain. Neprilysin, a major endopeptidase involved in proteolysis-related degradation of Aβ in the brain, is important in the clearance of Aβ [19]. In the present study, we found that the expression and activities of neprilysin were decreased in the Aβ_1–42_-infused ERα knockout mice. It is noteworthy that the activity of neprilysin is lower in AD patients [5, 10] and in ovaritectomized animals [20]. Moreover, neprilysin has two EREs, and its activity can be elevated by estrogen [21]. In present study, RAGE expression in the brain has no significant difference between ERα WT with ERα KO mice. Similar to RAGE, expressions of LRP-1 and MMP-9 have also no significant difference (data not shown). But, expression of LRP-1 was significantly decreased in ERα knockout mice liver. The rapid peripheral clearance of Aβ is mediated mainly by hepatic LRP-1 [22]. Reduced hepatic LRP-1 levels are associated with decreased peripheral Aβ clearance [23]. These data indicate that Aβ peptide degradation from the brain was prevented in the Aβ_1–42_-infused ERα knockout mice through a decrease of neprilysin activity. Expression of LRP-1, a clearance receptor protein, was also decreased in the Aβ_1–42_-infused ERα knockout mice liver. It should be known that down-regulation of LRP-1 in older mice is associated with Aβ accumulation in AD brains [24]. In our previous study, an AD mouse (APPsw mice) brain showed lower expression level of LRP-1 compared to a non-transgenic mouse brain [25]. We also found that MMP-9 was lowered in the Aβ_1–42_-infused ERα knockout mice. It was reported that estrogen activates MMP-9 thus increasing Aβ degradation [26]. Several studies have demonstrated that estrogen could stimulate Aβ degradation enzymes such as MMP in vivo and in vitro systems [26–28] as well as scavenger enzyme [29]. These data indicate that prevention systemic elimination through liver LRP-1 and MMP-9 could play at least a role in the increase of Aβ accumulation. Our present study suggests that impairment of multiple clearance mechanisms is involved in the accumulation of Aβ in the Aβ_1–42_-infused ERα knockout mice brain, thus affecting worsened memory impairment.

Astrocyte cells are important for the development of AD because they are highly reactive to environmental changes, such as oxidative stress and neuroinflammation [30]. Higher activation of astrocytes releases many soluble factors such as ROS, NO, PGs, TNF-α, and IL-1β which are critical for neuronal cell death [31]. Several studies also reported that Aβ can directly activate astrocytes [27, 32], which could contribute to the Aβ-induced neuronal cell death in vitro and in vivo [33]. Thus, worsened memory impairment in Aβ_1–42_-infused ERα knockout mice by accumulation of Aβ could be related with neuronal cell death via increased astrogliosis and neuroinflammation. In the present study, activation of astrocytes was greatly increased in the Aβ_1–42_-infused ERα knockout compared to the Aβ_1–42_-infused C57BL/6 wild-type mice. The activated astrocytes are predominant features of the inflammatory response in the brain of AD and contribute to neuronal cell death. We also found higher elevation of neuroinflammation evidenced by increased expression of iNOS and Cox-2, accompanied with elevation of GFAP expression. In response to brain injury, astrocytes become reactive and express various inflammatory mediators that play important roles in the secondary injury causing neuronal cell death. In this regard, we found that the expression of pro-apoptotic proteins such as Bax and caspase-3 was increased in the Aβ_1–42_-infused ERα knockout mice brain. It was also found that neuronal cell death was greatly increased in the Aβ_1–42_-infused ERα knockout mice brain compared to the Aβ_1–42_-infused C57BL/6 mice brain. These data indicate that accumulation of Aβ in ERα knockout mice resulted in the activation of astrocyte and neuroinflammation, thus causing neuronal cell death leading to worsened memory impairment. Although there is clear significance of ERα and ERβ in the protective role of estrogen in the AD development [5, 10, 20], the Women’s Health Initiative found that estrogen therapy on cognitive function for post-menopausal women is no longer a benefit for treatment [3, 4]. Thus, clearer mechanistic roles of estrogen in the development of AD should be further studied.

In conclusion, our data showed that infusion of Aβ_1–42_ in the mice brain of ERα knockout definitely decreases memory functions via an increase of astrogliosis and neuroinflammation-related neuronal cell death through diminished Aβ elimination.

## Electronic Supplementary Material

Below is the link to the electronic supplementary material.

Supplementary Figure 1 Effect of ERα knockout on RAGE levels in mouse brain, and MMP-9 and LRP-1 levels in mouse Liver, Immunostaining of RAGE in the cortex and hippocampus was performed 30 μm-thick sections of mice brain incubated with anti-RAGE primary antibodies and the biotinylated secondary antibody. The representative stained tissues were viewed with a microscope (×50 or 200) **a**. Tissue lysates from mice brain were probed with RAGE antibody, respectively. **b**, Tissue lysates from mice liver were probed with MMP-9 and LRP-1 antibody, respectively **c**. Experiments were performed from three mice brains. β-Actin levels were measured for the confirmation of equal amount of protein loading. (GIF 160 kb)

High Resolution Image (tiff 252 kb)

Supplementary Figure 2 .Effect of ERα knockout on expression and activity of β-secretase in mouse brain, The expression of APP and BACE1 were detected by western blotting using specific antibodies in the mouse brain. Each blot is representative of three experiments **a**. The activity of β-secretase was investigated using assay kit as described **b**. Values measured from each group of mice were calibrated by the amount of protein and expressed as mean ± SEM (*n* = 8 mice). * Significant difference between saline-infused to Aβ-infused mice (*P* < 0.05). # Significant difference between Aβ-infused C57BL/6 wild type mice to Aβ-infused ERα KO mice (*P* < 0.05). (GIF 98 kb)

High Resolution Image (tiff 105 kb)

Supplementary Figure 3 Location of osmotic pump. Infusion region was inserted unilaterally − 1.0 mm anterior/posterior, + 0.5 mm medial/lateral and − 2.5 mm dorsal/ventral. The pumps were fixed under skin of mice's back. The pump contents were released over a period of 2 weeks consisting of 300 pmol aggregated Aβ_1–42_ dissolved in sterile saline (0.9 % NaCl) for each pump (GIF 143 kb)

High Resolution Image (tiff 134 kb)

## Abbreviations

Aβ: Amyloid-beta
AD: Alzheimer’s disease
ANOVA: Analysis of variance
APP: Amyloid precursor protein
BACE: β-Secretase
Cox-2: Cyclooxygenase-2
ELISA: Enzyme-linked immunosorbent assay
ER: Estrogen receptor
EREs: Estrogen response elements
GFAP: Glial fibrillary acidic protein
IL: Interleukin
ICV: Intracerebroventricular
iNOS: Inducible nitric oxide synthase
KO: Knockout
LRP: Lipoprotein receptor-related protein
MMPs: Matrix metalloproteinases
NEP: Neprilysin
NO: Nitric oxide
PBS: Phosphate-buffered saline
PG: Prostaglandin
RAGE: Receptor for advanced glycation end products
ROS: Reactive oxygen species
TNF: Tumor necrosis factor

## References

[1] Shughrue PJ, Lane MV, Merchenthaler I (1997). Comparative distribution of estrogen receptor-alpha and -beta mRNA in the rat central nervous system. J Comp Neurol.

[2] Janicki SC, Schupf N (2010). Hormonal influences on cognition and risk for Alzheimer’s disease. Curr Neurol Neurosci Reports.

[3] Anderson GL, Chlebowski RT, Rossouw JE, Rodabough RJ, McTiernan A, Margolis KL, Aggerwal A, David Curb J, Hendrix SL, Allan Hubbell F, Khandekar J, Lane DS, Lasser N, Lopez AM, Potter J, Ritenbaugh C (2006). Prior hormone therapy and breast cancer risk in the Women’s Health Initiative randomized trial of estrogen plus progestin. Maturitas.

[4] Espeland MA, Hogan PE, Fineberg SE, Howard G, Schrott H, Waclawiw MA, Bush TL (1998). Effect of postmenopausal hormone therapy on glucose and insulin concentrations. PEPI Investigators. Postmenopausal Estrogen/Progestin Interventions. Diabetes care.

[5] Henderson VW (2010). Action of estrogens in the aging brain: dementia and cognitive aging. Biochim Biophys Acta.

[6] Levin-Allerhand J, McEwen BS, Lominska CE, Lubahn DB, Korach KS, Smith JD (2001). Brain region-specific up-regulation of mouse apolipoprotein E by pharmacological estrogen treatments. J Neurochem.

[7] Xu H, Gouras GK, Greenfield JP, Vincent B, Naslund J, Mazzarelli L, Fried G, Jovanovic JN, Seeger M, Relkin NR, Liao F, Checler F, Buxbaum JD, Chait BT, Thinakaran G, Sisodia SS, Wang R, Greengard P, Gandy S (1998). Estrogen reduces neuronal generation of Alzheimer beta-amyloid peptides. Nat Med.

[8] Wang DS, Dickson DW, Malter JS (2006). beta-Amyloid degradation and Alzheimer’s disease. J Biomed Biotechnol.

[9] Yasojima K, Akiyama H, McGeer EG, McGeer PL (2001). Reduced neprilysin in high plaque areas of Alzheimer brain: a possible relationship to deficient degradation of beta-amyloid peptide. Neurosci Lett.

[10] Hellstrom-Lindahl E, Ravid R, Nordberg A (2008). Age-dependent decline of neprilysin in Alzheimer’s disease and normal brain: inverse correlation with A beta levels. Neurobiol Aging.

[11] Huang J, Guan H, Booze RM, Eckman CB, Hersh LB (2004). Estrogen regulates neprilysin activity in rat brain. Neurosci Lett.

[12] Foster TC (2012). Role of estrogen receptor alpha and beta expression and signaling on cognitive function during aging. Hippocampus.

[13] Nabeshima T, Nitta A (1994). Memory impairment and neuronal dysfunction induced by beta-amyloid protein in rats. Tohoku J Exp Med.

[14] Morris R (1984). Developments of a water-maze procedure for studying spatial learning in the rat. J Neurosci Methods.

[15] Limon D, Diaz A, Hernandez M, Fernandez GJ, Torres-Martinez AC, Perez-Severiano F, Rendon-Huerta EP, Montano LF, Guevara J (2012). Neuroprotective effect of the aminoestrogen prolame against impairment of learning and memory skills in rats injected with amyloid-beta-25–35 into the hippocampus. Eur J Pharmacol.

[16] Xu WJ, Xu F, Anderson ME, Kotarba AE, Davis J, Robinson JK, Van Nostrand WE (2013). Cerebral microvascular rather than parenchymal amyloid-beta protein pathology promotes early cognitive impairment in transgenic mice. J Alzheimers Dis: JAD.

[17] Hardy J, Selkoe DJ (2002). The amyloid hypothesis of Alzheimer’s disease: progress and problems on the road to therapeutics. Science.

[18] Rune GM, Wehrenberg U, Prange-Kiel J, Zhou L, Adelmann G, Frotscher M (2002). Estrogen up-regulates estrogen receptor alpha and synaptophysin in slice cultures of rat hippocampus. Neuroscience.

[19] Nishida Y, Ito S, Ohtsuki S, Yamamoto N, Takahashi T, Iwata N, Jishage K, Yamada H, Sasaguri H, Yokota S, Piao W, Tomimitsu H, Saido TC, Yanagisawa K, Terasaki T, Mizusawa H, Yokota T (2009). Depletion of vitamin E increases amyloid beta accumulation by decreasing its clearances from brain and blood in a mouse model of Alzheimer disease. J Biol Chem.

[20] Li R, He P, Cui J, Staufenbiel M, Harada N, Shen Y (2013). Brain endogenous estrogen levels determine responses to estrogen replacement therapy via regulation of BACE1 and NEP in female Alzheimer’s transgenic mice. Mol Neurobiol.

[21] Sinopoli KJ, Floresco SB, Galea LA (2006). Systemic and local administration of estradiol into the prefrontal cortex or hippocampus differentially alters working memory. Neurobiol Learn Mem.

[22] Tamaki C, Ohtsuki S, Iwatsubo T, Hashimoto T, Yamada K, Yabuki C, Terasaki T (2006). Major involvement of low-density lipoprotein receptor-related protein 1 in the clearance of plasma free amyloid beta-peptide by the liver. Pharm Res.

[23] Hickman SE, Allison EK, El Khoury J (2008). Microglial dysfunction and defective beta-amyloid clearance pathways in aging Alzheimer’s disease mice. J Neurosci: Off J Soc Neurosci.

[24] Pascale CL, Miller MC, Chiu C, Boylan M, Caralopoulos IN, Gonzalez L, Johanson CE, Silverberg GD (2011). Amyloid-beta transporter expression at the blood-CSF barrier is age-dependent. Fluids and Barriers of the CNS.

[25] Lee YJ, Choi DY, Lee YK, Lee YM, Han SB, Kim YH, Kim KH, Nam SY, Lee BJ, Kang JK, Yun YW, Oh KW, Hong JT (2012). 4-*O*-Methylhonokiol prevents memory impairment in the Tg2576 transgenic mice model of Alzheimer’s disease via regulation of beta-secretase activity. J Alzheimers Dis: JAD.

[26] Merlo S, Sortino MA (2012). Estrogen activates matrix metalloproteinases-2 and -9 to increase beta amyloid degradation. Mol Cell Neurosci.

[27] Zhao J, O’Connor T, Vassar R (2011). The contribution of activated astrocytes to Aβ production: implications for Alzheimer’s disease pathogenesis. J Neuroinflammation.

[28] Liang K, Yang L, Yin C, Xiao Z, Zhang J, Liu Y, Huang J (2010). Estrogen stimulates degradation of beta-amyloid peptide by up-regulating neprilysin. J Biol Chem.

[29] Tang YP, Haslam SZ, Conrad SE, Sisk CL (2004). Estrogen increases brain expression of the mRNA encoding transthyretin, an amyloid beta scavenger protein. J Alzheimers Dis: JAD.

[30] Hall ED, Oostveen JA, Gurney ME (1998). Relationship of microglial and astrocytic activation to disease onset and progression in a transgenic model of familial ALS. Glia.

[31] Gibson RM, Rothwell NJ, Le Feuvre RA (2004). CNS injury: the role of the cytokine IL-1. Vet J.

[32] Schubert D, Soucek T, Blouw B (2009). The induction of HIF-1 reduces astrocyte activation by amyloid beta peptide. Eur J Neurosc.

[33] Butterfield DA, Boyd-Kimball D (2004). Amyloid beta-peptide (1–42) contributes to the oxidative stress and neurodegeneration found in Alzheimer disease brain. Brain Pathol.

