# Supplementary figures and images for: Memory Impairment in Estrogen Receptor α Knockout Mice Through Accumulation of Amyloid-β Peptides
Source: Mol Neurobiol. 2014 Aug 17;52(1):176–86. doi: 10.1007/s12035-014-8853-z (PMC4510915; doi:10.1007/s12035-014-8853-z)

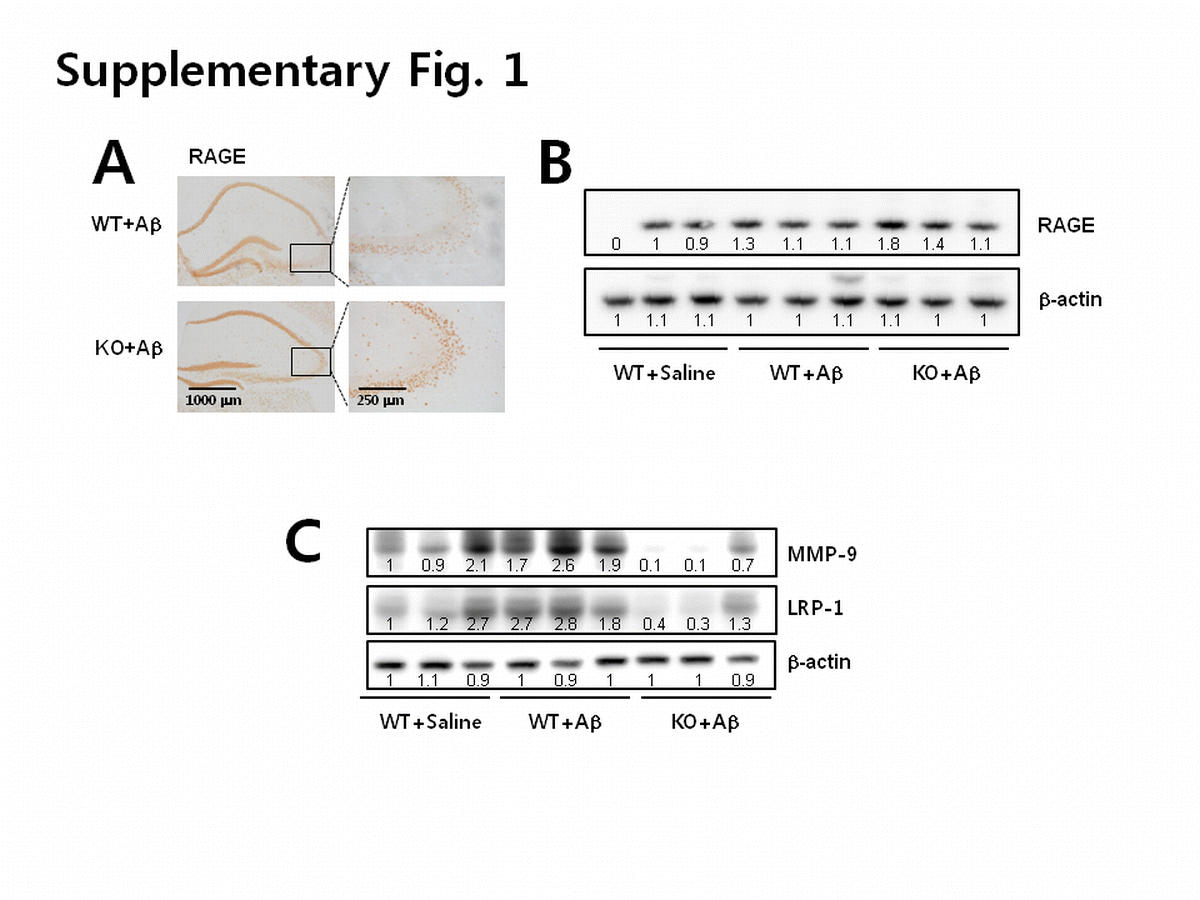

Supplement: Supplementary file 1 — Effect of ERα knockout on RAGE levels in mouse brain, and MMP-9 and LRP-1 levels in mouse Liver, Immunostaining of RAGE in the cortex and hippocampus was performed 30 μm-thick sections of mice brain incubated with anti-RAGE primary antibodies and the biotinylated secondary antibody. The representative stained tissues were viewed with a microscope (×50 or 200) a. Tissue lysates from mice brain were probed with RAGE antibody, respectively. b, Tissue lysates from mice liver were probed with MMP-9 and LRP-1 antibody, respectively c. Experiments were performed from three mice brains. β-Actin levels were measured for the confirmation of equal amount of protein loading. (GIF 160 kb) [file 12035_2014_8853_Fig6_ESM.gif]

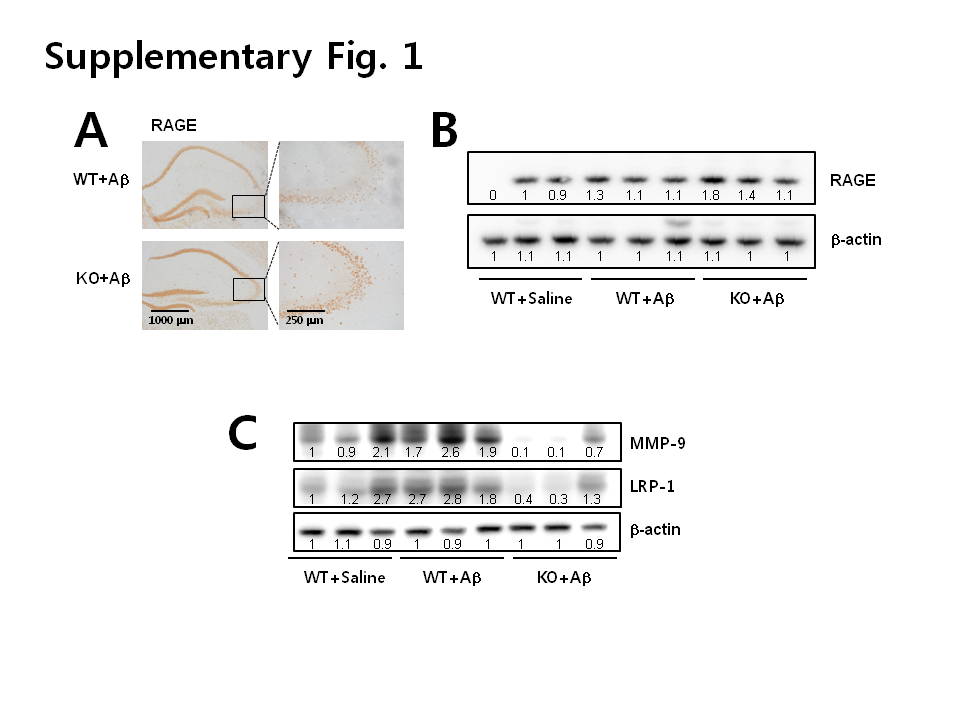

Supplement: Supplementary file 2 — High Resolution Image (tiff 252 kb) [file 12035_2014_8853_MOESM1_ESM.tif]

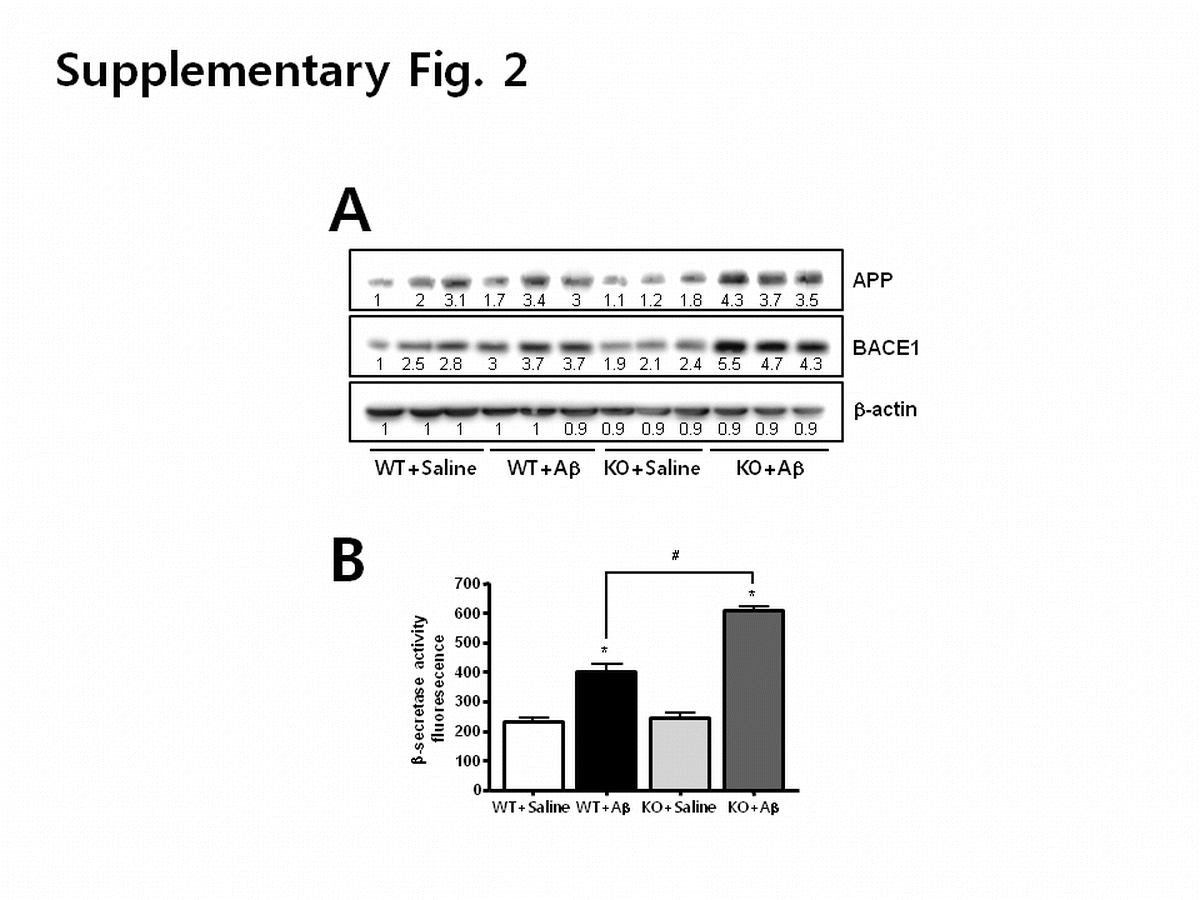

Supplement: Supplementary file 3 — .Effect of ERα knockout on expression and activity of β-secretase in mouse brain, The expression of APP and BACE1 were detected by western blotting using specific antibodies in the mouse brain. Each blot is representative of three experiments a. The activity of β-secretase was investigated using assay kit as described b. Values measured from each group of mice were calibrated by the amount of protein and expressed as mean ± SEM (n = 8 mice). * Significant difference between saline-infused to Aβ-infused mice (P < 0.05). # Significant difference between Aβ-infused C57BL/6 wild type mice to Aβ-infused ERα KO mice (P < 0.05). (GIF 98 kb) [file 12035_2014_8853_Fig7_ESM.gif]

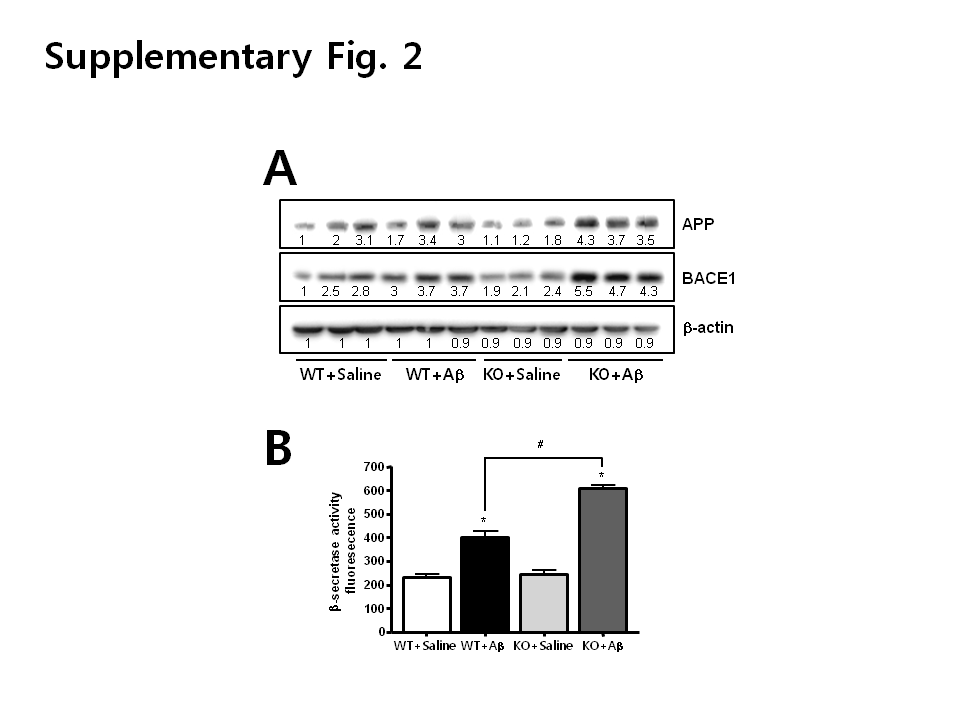

Supplement: Supplementary file 4 — High Resolution Image (tiff 105 kb) [file 12035_2014_8853_MOESM2_ESM.tif]

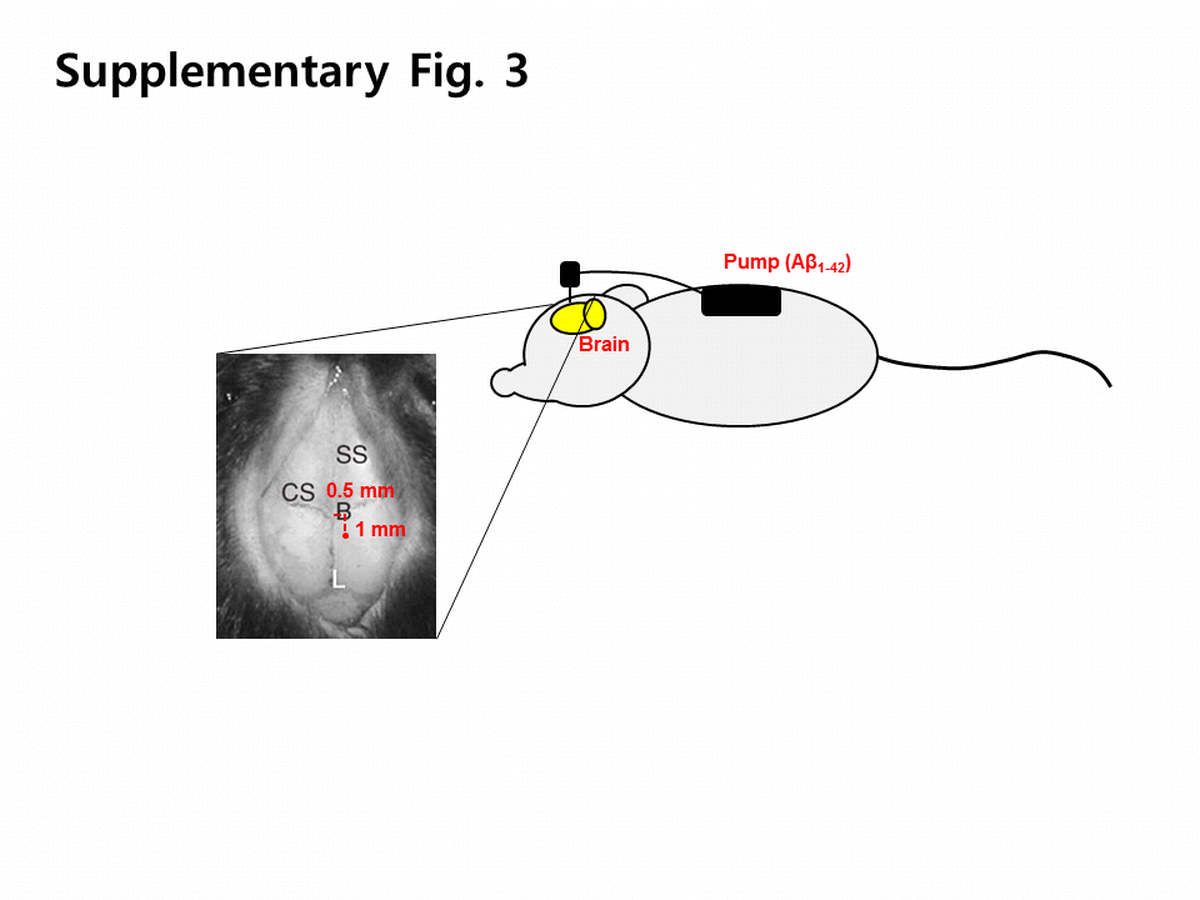

Supplement: Supplementary file 5 — Location of osmotic pump. Infusion region was inserted unilaterally − 1.0 mm anterior/posterior, + 0.5 mm medial/lateral and − 2.5 mm dorsal/ventral. The pumps were fixed under skin of mice's back. The pump contents were released over a period of 2 weeks consisting of 300 pmol aggregated Aβ1–42 dissolved in sterile saline (0.9 % NaCl) for each pump (GIF 143 kb) [file 12035_2014_8853_Fig8_ESM.gif]

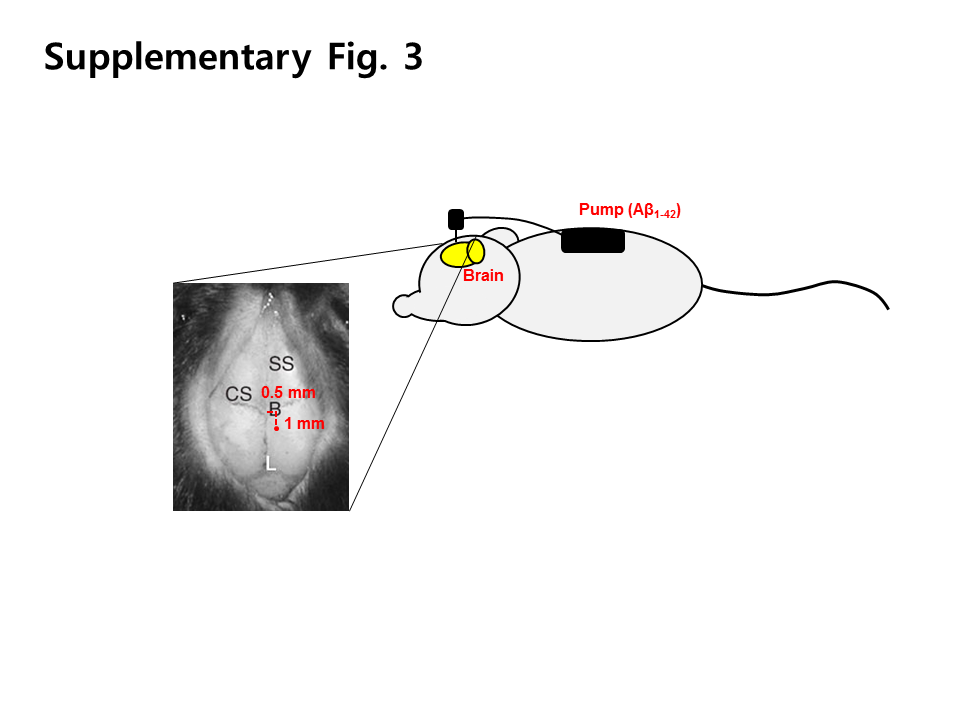

Supplement: Supplementary file 6 — High Resolution Image (tiff 134 kb) [file 12035_2014_8853_MOESM3_ESM.tif]
